# Giant Cervicothoracic Cystic Lymphangioma in a Neonate: Clinical Challenges and Management Considerations

**DOI:** 10.1055/a-2739-1840

**Published:** 2025-11-17

**Authors:** Marina A. Bustamante-Ordoñez, Gianelli S. Curi-Vilchez, Eduardo Marchand-Bayona, Carlos Zavaleta-Corvera, Angel Samanez-Obeso

**Affiliations:** 1 Faculty of Medicine, Universidad Científica del Sur, Lima, Perú; 2 Subunidad de Atención Integral Especializada de Cirugía Neonatal y Pediátrica Compleja, Instituto Nacional de Salud del Niño San Borja, Lima, Perú

**Keywords:** lymphangioma, lymphatic malformations, sclerotherapy, surgical complications

## Abstract

**Background:**

Lymphangiomas are benign vascular malformations of the lymphatic system, commonly affecting the head and neck. Neonatal cases pose significant clinical and surgical challenges.

**Case Presentation:**

We report a premature female neonate (35 weeks of gestation) with a large cervicothoracic cystic lymphangioma (8 × 7 cm), causing respiratory distress and vascular compression. Imaging revealed an extensive lymphatic malformation (160 × 67 × 87 mm), affecting the sternocleidomastoid muscle, salivary glands, and adjacent vasculature. Surgical resection achieved 90% tumor removal while preserving neurovascular structures. Postoperative complications included transient neuromotor deficit of the right upper limb and *Candida lusitaniae* sepsis. Despite intensive care, tumor progression led to respiratory failure, and the patient succumbed 12 days' postsurgery.

**Discussion:**

Extensive cervicothoracic lymphangiomas in neonates require complex management. Imaging aids in surgical planning, delineating tumor extent, and complications. While surgical resection is the standard treatment, it carries risks in neonates with large lesions. Sclerotherapy has shown efficacy in macrocystic lymphangiomas but remains debated for extensive neonatal cases. A multidisciplinary approach is crucial to optimize outcomes.

**Conclusion:**

Neonatal cervicothoracic lymphangiomas pose significant challenges. Surgery remains primary, but sclerotherapy may be considered in selected cases. Multidisciplinary management is essential to improve prognosis and reduce morbidity.

## Introduction

Lymphangiomas represent a group of benign vascular malformations of the lymphatic system, characterized by the proliferation of dilated cystic lymphatic vessels with a notable ability to infiltrate adjacent tissues and structures. 1 2 They originate from an abnormal development of the lymphatic system due to a failed communication between primitive lymphatic channels and the venous drainage system, leading to lymph accumulation and cyst formation. 3 4

The predominant location of lymphangiomas is the head and neck, accounting for approximately 75 to 80% of cases. The cervical region is the most affected, especially in the posterior triangle of the neck, due to the high density of lymphatic structures in this area. 3 Based on their location, lymphangiomas can be classified as superficial, deep, or mixed, with relevant clinical and surgical implications.

Approximately 90% of lymphangiomas are diagnosed in children under the age of 2, with presentation in adults being a rare event. 5 Clinically, these lesions present as soft, slow-growing, and compressible masses. However, in some cases, they may cause severe complications such as airway compression, esophageal obstruction, secondary infection, or intralesional hemorrhage.

Imaging modalities play a fundamental role in diagnosis. Computed tomography (CT) is the imaging technique of choice, as it allows a detailed assessment of the size, extent, and relationship with adjacent structures, making it essential for surgical planning. 6 However, in neonates and infants, ultrasound is useful as an initial tool due to its ability to differentiate between liquid and solid components within the lesion. In selected cases, magnetic resonance imaging (MRI) may be necessary to evaluate deep tissue infiltration.

The treatment of choice for cystic lymphangiomas is complete surgical resection, as it reduces the likelihood of recurrence. However, excision can be challenging when the lesion involves major neurovascular structures, increasing the risk of nerve injury, hemorrhage, or postoperative dysfunction. 6 In cases where surgery is not feasible or poses a high risk, alternative therapies such as sclerotherapy with sclerosing agents (bleomycin, OK-432, doxycycline) have been explored, with variable outcomes depending on the size and location of the lymphangioma.

This case report describes the clinical course, diagnosis, treatment, and complications of a neonate with a cervicothoracic cystic lymphangioma. The aim is to provide updated information on the management of this rare condition, highlight its clinical and surgical challenges, and encourage future research in the field of pediatric and neonatal surgery.

## Case Presentation

We presented the case of a premature female neonate, born via vaginal delivery at 35 weeks of gestation, with a birth weight of 2,565 g and an Apgar score of 8/9 at 1 and 5 minutes, respectively. At 5 days of life, she was referred to a higher-complexity hospital due to the presence of a cervicothoracic tumor, with no relevant family history of congenital malformations or similar pathologies.

The mother had five prenatal checkups and presented with a urinary tract infection during the second trimester of pregnancy, which was treated with antibiotics. A prenatal ultrasound in the third trimester identified a lateral mass in the right hemithorax, suggesting the possibility of a congenital malformation of lymphatic or vascular origin.

**Fig. 1:**
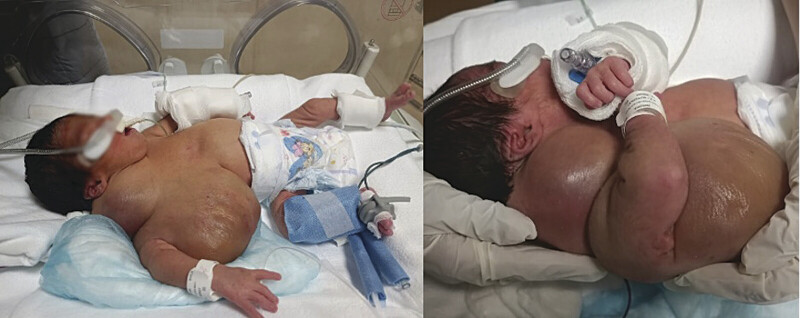
Clinical photograph of a neonate with an extensive cervicothoracic cystic lymphangioma; a large volume mass in the right lateral neck region and upper thorax.

Upon admission to the neonatal emergency department, the patient was awake, reactive, with adequate perfusion and spontaneous ventilation. She had no fever or signs of neurological focalization. Physical examination revealed weak crying, mild jaundice (zone 2 on the Kramer scale), and a cervical mass measuring 8 × 7 cm, with well-defined borders, soft consistency, and a regular surface ( Fig. 1 ). Additionally, a submaxillary mass with a shiny and indurated surface extended to the costal margin. There was slight respiratory difficulty but no evident signs of acute respiratory failure. Generalized edema was observed, with moist gauze and serous secretion in the thoracic region. The right hand showed slight pallor, with present and symmetrical peripheral pulses. The abdomen was soft, globular, with no masses or hepatosplenomegaly.

Given the clinical presentation, cervicothoracic lymphangioma with possible respiratory involvement and neonatal sepsis were suspected, leading to the request for auxiliary laboratory tests and imaging studies.

Laboratory findings showed a hematocrit of 54.4%, platelet count of 301,000 µL, prothrombin time of 15.5 seconds, international normalized ratio of 1.17, and activated partial thromboplastin time of 48.8 seconds. Liver profile tests revealed a total bilirubin level of 9.25 mg/dL, direct bilirubin of 0.55 mg/dL, and albumin of 3.31 g/dL. Blood cultures were negative, and levels of C-reactive protein, arterial blood gas analysis, and thyroid profile were normal.

**Fig. 2:**
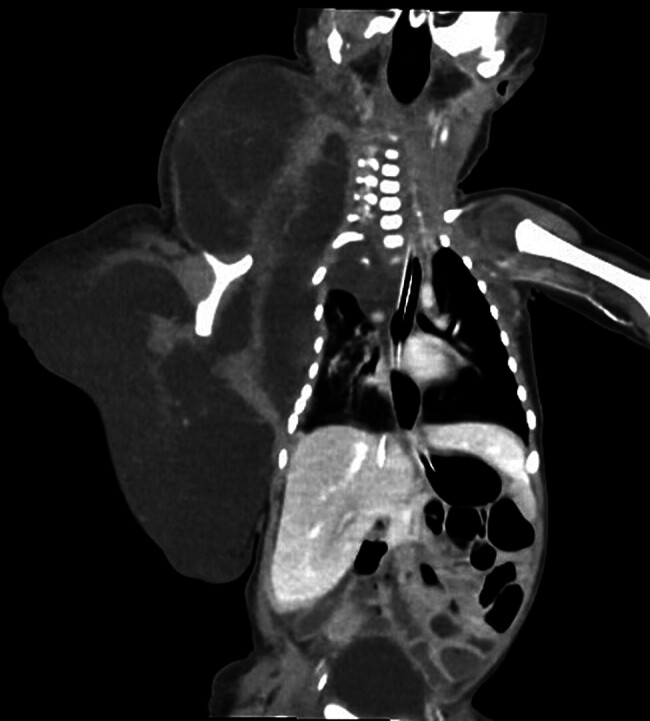
Chest computed tomography (CT) showing a large multiloculated cystic lesion in the right cervicothoracic region, displacing adjacent structures and compressing the sternocleidomastoid muscle, parotid, and submandibular glands. Subsegmental atelectasis is observed in the lower right lung lobe, with no evidence of bone involvement. Findings are consistent with a giant cystic lymphangioma.

A Doppler ultrasound showed decreased blood pressure in the right brachial artery, consistent with vascular compression. A contrast-enhanced thoracic CT scan and cervical CT angiography (CTA) revealed an extensive lymphangioma with well-defined margins, originating in the subcutaneous tissue of the right cervical, thoracic, and abdominal regions, measuring approximately 160 × 67 × 87 mm. Displacement and compression of the right sternocleidomastoid muscle, parotid gland, and submandibular gland were observed, along with subsegmental atelectasis in the lower right lung lobe, with no apparent involvement of neural structures ( Fig. 2 ).

Based on these findings, surgical resection of the tumor was planned. During the procedure, general anesthesia was administered, and a wide vertical axillary incision was made, identifying a multilocular cystic tumor with a moderate amount of yellowish serous secretion. A meticulous dissection was performed using electrocautery and Ligasure, preserving neurovascular structures, achieving 90% resection of the tumor, extending to the posterior region of the right scapula. A Penrose drain was placed in the surgical bed. Subsequently, a cervical lozenge-shaped incision was made, with complete lymphangioma resection, closing in layers with Vicryl 4/0 and Nylon 5/0.

**Fig. 3:**
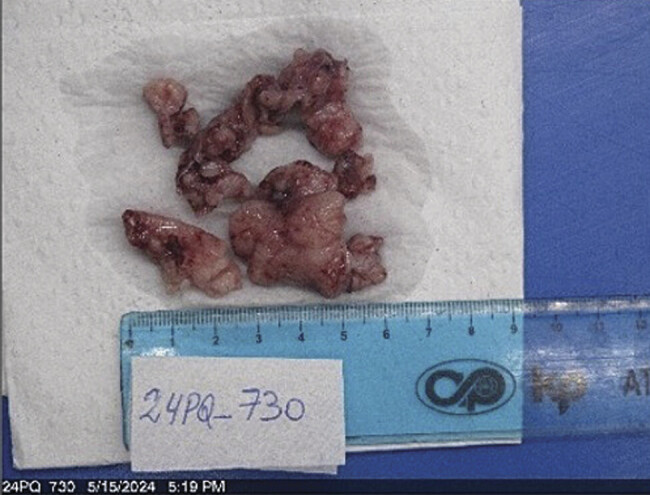
Macroscopic image showing multilobulated soft tissue fragments, ranging in color from pink to reddish, with an irregular surface and cystic appearance. Some areas contain serohematic fluid, and the overall consistency is predominantly soft. The approximate fragment dimensions range from 2 to 4 cm, consistent with the diagnosis of cystic lymphangioma.

The macroscopic pathological analysis described a multilocular cystic tumor in the right lateral cervical region, approximately 8 × 8 cm in size, with yellowish serous-hemorrhagic fluid content, extending from the midline to the posterior region, clavicle, and scapula ( Fig. 3 ).

**Fig. 4:**
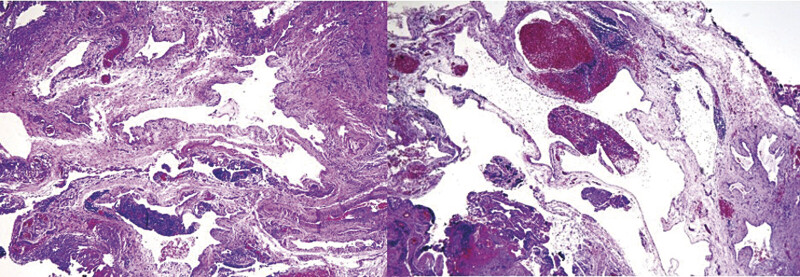
Microscopic pathology reveals dilated vascular spaces lined by small, soft endothelial cells, consistent with a lymphatic vascular malformation.

**Fig. 5:**
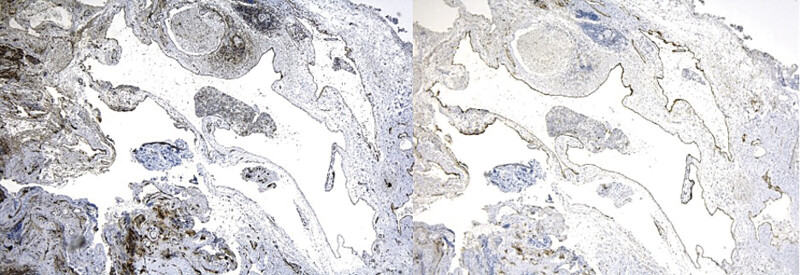
Left: immunohistochemistry positive for CD31. Right: immunohistochemistry positive for podoplanin.

Microscopically, dilated vascular spaces lined by small endothelial cells with oval, hyperchromatic, and flattened nuclei were observed, along with focal lymphoid infiltration. Immunohistochemical analysis confirmed the diagnosis of cervical cystic lymphangioma, with positive results for CD31 and podoplanin and negative for WT-1 ( Figs. 4 , 5 ). Histologically, there was no evidence of cytological atypia, mitotic figures, or tissue disorganization suggestive of malignancy. The lesion exhibited benign features consistent with a lymphatic malformation (LM), with no dysplastic or neoplastic changes.

During the initial postoperative evaluation, there was no movement in the right upper limb, raising suspicion of neuromuscular involvement. Follow-up imaging studies were performed, but no structural lesions were evident.

Six days after surgery, the patient showed signs of neonatal sepsis. A follow-up complete blood count revealed severe leukopenia (1,000 leukocytes/mm ^3^ ), and urine culture was positive for *Candida lusitaniae* . Treatment was initiated with vancomycin, meropenem, and fluconazole, but the clinical condition continued to deteriorate progressively.

Despite intensive medical management, the tumor showed progressive growth, leading to airway collapse and infiltration of adjacent structures. After multiple discussions with the medical team, the family decided not to pursue additional surgical interventions.

The patient ultimately passed away 12 days after surgery due to severe respiratory failure secondary to tumor growth.

## Discussion

### Clinical Presentation and Differential Diagnosis

Cystic lymphangioma is a benign malformation of the lymphatic system, characterized by the abnormal dilation of lymphatic ducts due to a failure in communication between the lymphatic and venous systems during embryonic development. 1 There are two main subtypes based on the size of the vascular space: simple lymphangiomas, composed of lymphatic vessels similar in caliber to capillaries, and cavernous lymphangiomas, which consist of dilated lymphatic channels with multiple endothelial layers. 2

According to the 2018 classification by the International Society for the Study of Vascular Anomalies, the lesion corresponds to a macrocystic LM, given the presence of multiple large cystic spaces with a benign endothelial lining. It falls under the category of simple LMs, which are classified as low-flow vascular anomalies.

These tumors can occur anywhere in the body, but the cervical region is the most affected, particularly the posterior triangle of the neck (75% of cases), followed by the submandibular triangle (20%), and other less common locations (5%) such as the mediastinum, retroperitoneum, and abdominal cavity. 3 In the presented case, the cervicothoracic location with infiltration of adjacent structures suggests an aggressive and expansive component, which is rare in neonates.

The differential diagnosis includes congenital and neoplastic lesions such as cystic teratoma, branchial cleft cyst, thyroid tumor, laryngocele, cervical neuroblastoma, lymphadenitis, lipoma, and other vascular malformations. 4 5 In this context, imaging evaluation is crucial to differentiate lymphangioma from other entities and determine its anatomical extent ( Table 1 ). 7 8

**Table 1:** Differential diagnosis of cervical masses

Characteristic	Cystic lymphangioma	Cervical teratoma	Thyroglossal duct cyst	Branchial cleft cyst
Origin	Lymphatic malformation	Germ cell tumor	Remnant of the thyroglossal duct	Remnant of the branchial cleft
Clinical presentation	Soft, compressible mass, positive transillumination	Solid mass, sometimes with calcifications	Midline mass, moves with swallowing	Lateral neck mass, may drain
Age of onset	Usually before 2 y of age	At birth or prenatal period	Childhood or adolescence	Childhood or adolescence
Diagnostic imaging	Multiloculated cysts on ultrasound/computed tomography	Heterogeneous mass with solid and cystic areas	Well-defined midline cyst	Well-defined lateral neck cyst
Treatment of choice	Surgical resection or sclerotherapy	Surgical resection	Surgical resection (Sistrunk procedure)	Surgical resection

### Importance of Imaging Studies in Diagnosis and Surgical Planning

Imaging studies are essential for establishing a definitive diagnosis and defining the therapeutic strategy. CT and MRI are the most used modalities. MRI is considered the preferred technique, as it allows for the evaluation of the tumor's internal composition and its relationship with critical structures. 6 However, in neonates and infants, contrast-enhanced CT remains highly useful, as in the case described, where CTA helped delineate the involvement of vascular and muscular structures.

In the presented patient, CTA revealed a large lymphangioma (160 × 67 × 87 mm), with compression of the sternocleidomastoid muscle, the parotid and submandibular glands, as well as subsegmental atelectasis in the lower right lung lobe. These findings were critical in the surgical decision-making process due to the potential risk of airway compromise and respiratory complications.

### Medical Treatment

Cervical cystic lymphangioma is a benign LM, and its therapeutic management is based on two main strategies: surgical resection and sclerotherapy. The choice of treatment depends on several factors, such as size, location, type, and the presence of compressive symptoms. Traditionally, surgery has been the treatment of choice, particularly for extensive lesions or those involving critical structures such as the airway or major blood vessels. 9 However, in neonates and infants, surgery presents significant challenges, as the procedure may involve complications such as hemorrhage, neurovascular injury, and lymphatic leakage, as well as requiring prolonged hospitalization and intensive postoperative care. 10 Although complete resection reduces the risk of recurrence, the associated morbidity has led to the search for less invasive alternatives.

Sclerotherapy has emerged as an effective and safe therapeutic option for macrocystic and mixed lymphangiomas, especially in pediatric patients. 11 In a study involving 15 patients with cystic lymphangioma, infiltration with OK-432 (Picibanil) achieved complete lesion reduction in 40% of cases, with significant improvement in another 33.4% and a low recurrence rate of 6.6%. 9 These findings suggest that sclerotherapy can be a viable alternative to surgery, particularly in neonatal patients where invasive procedures are best avoided. One of its main advantages is a faster and mostly outpatient recovery, with a favorable safety profile. 12 Unlike surgery, where complications include nerve damage and extensive scarring, the primary adverse effects of sclerotherapy are local inflammation and, in rare cases, transient infection. 13

Although sclerotherapy has shown good results in predominantly cystic lymphangiomas, it is not always effective in microcystic lesions, which may require complementary surgical treatment. 10 The decision between surgery and sclerotherapy should be individualized, considering the location, lesion size, and resource availability. In neonates, sclerotherapy is emerging as a first-line option due to its lower risk of complications and its ability to reduce the need for surgery in selected cases. 11 However, in large lesions with deep infiltration, surgical resection remains the standard treatment for achieving definitive resolution. 14 A multidisciplinary approach involving pediatric surgeons, radiologists, and neonatologists is recommended to determine the most appropriate therapeutic strategy for each patient 5 12 14 ( Table 2 ).

**Table 2:** Comparative summary of therapeutic options

Aspect	Surgical resection	Sclerotherapy (OK-432, bleomycin)
Indications	Large or symptomatic lesions	Microcystic lesions or patients unfit for surgery
Success rate	High if resection is complete	Variable; better in macrocystic lesions
Complications	Injury to adjacent structures, scarring	Local inflammation, infection
Recurrence	Low with complete resection	Possible, requires multiple sessions
Recovery time	Prolonged, depends on extent	Generally shorter, outpatient in some cases

### Surgical Challenges and Postoperative Management

The treatment of choice for symptomatic cervicothoracic lymphangiomas is complete surgical resection, with the goal of preventing recurrences and minimizing complications. 15 However, surgery is particularly challenging when the lesion infiltrates critical structures such as nerves, blood vessels, or the airway, increasing the risk of neurological injury, hemorrhage, or respiratory complications.

In this case, a broad resection of the lymphangioma was achieved without intraoperative complications. However, in the immediate postoperative period, a neuromotor deficit in the right upper limb was observed, suggesting involvement of the brachial plexus due to mass effect or surgical manipulation. This type of alteration has been described in the literature as an uncommon but relevant complication in cervical lymphangioma surgeries. 16

### Postoperative Complications and Clinical Course

Despite an initially stable course, the patient developed neonatal sepsis 6 days after surgery, with severe leukopenia (1,000 leukocytes/mm ^3^ ) and a urine culture positive for *C. lusitaniae* . This complication is unusual and may be related to predisposing factors such as transient postoperative immunosuppression, impaired lymphatic drainage, or prolonged hospitalization. 17

Antimicrobial management included vancomycin, meropenem, and fluconazole, but the patient continued to deteriorate. After multiple medical board discussions, the family opted against further surgical interventions, leading to the progression of the lymphangioma and its infiltration into adjacent structures, ultimately resulting in airway collapse and the patient's death.

### Considerations on the Use of Sclerotherapy and Therapeutic Alternatives

In recent years, sclerotherapy has gained popularity as a first-line treatment for lymphangiomas, especially in lesions that are difficult to resect. Agents such as bleomycin and OK-432 have demonstrated efficacy in reducing tumor size without the need for surgery in some cases. 14 18 However, the success rate varies, and adverse reactions include pain, local inflammation, and a risk of fibrosis.

In this case, sclerotherapy was not initially considered due to the tumor's large size and involvement of vital structures. However, the clinical outcome highlights the need for an individualized assessment of treatment, considering combined therapies in high-risk surgical situations.

### Final Considerations and Lessons Learned

This case highlights several key aspects in the management of giant lymphangiomas in neonates:

1. The importance of early diagnosis, as prenatal or early childhood identification can facilitate less invasive therapeutic strategies.
2. The crucial role of imaging evaluation, where CTA and MRI allow for lesion characterization and anticipation of possible surgical complications.
3. The challenges of surgical management, particularly in extensive tumors with infiltration of critical structures.
4. Considerations regarding sclerotherapy, which has demonstrated efficacy but must be individualized based on the location and extent of the lymphangioma.
5. The importance of a multidisciplinary approach, as a team of pediatric surgeons, radiologists, neonatologists, and intensive care specialists can optimize outcomes and reduce complications.

## Conclusion

The management of cervical cystic lymphangioma in neonates requires an individualized evaluation. While surgery remains the definitive treatment, sclerotherapy is a viable alternative in selected cases. Postoperative complications, such as neonatal sepsis, highlight the importance of a multidisciplinary approach and combined therapeutic strategies to improve prognosis.
